# Obesity in Type 1 Diabetes: Moving Beyond the “Lean” Disease Paradigm to Understand Risk, Complications, and Treatment

**DOI:** 10.1007/s13679-026-00693-9

**Published:** 2026-02-21

**Authors:** Anastasios Tentolouris, Theocharis Koufakis, Evangelos Fousteris

**Affiliations:** 1 First Department of Propaedeutic Internal Medicine and Diabetes Center, School of Medicine, National and Kapodistrian University of Athens, Laiko General Hospital, 17 Agiou Thoma Street, Athens, 11527 Greece; 2 Second Propaedeutic Department of Internal Medicine, Hippokration General Hospital, Aristotle University of Thessaloniki, Thessaloniki, 54642 Greece; 3 Mediterranean Diabetes and Obesity Clinics, Athens, 17561 Greece

**Keywords:** Cardiovascular complications, Microvascular complications, Obesity, Obesity management, Type 1 diabetes mellitus, Weight management

## Abstract

**Purpose of Review:**

Overweight and obesity are increasingly common in people with type 1 diabetes mellitus (T1DM). This narrative review synthesizes current evidence on the epidemiology and mechanisms linking excess adiposity with T1DM, the obesity-associated burden of complications, and approaches to obesity management in this population.

**Recent Findings:**

Excess adiposity is now frequent in people with T1DM, approaching general-population prevalence, and obesity may also increase the risk of incident T1DM. In T1DM, weight gain reflects intersecting drivers, including intensive insulin therapy, hypoglycemia-related compensatory intake and activity avoidance, obesity-related insulin resistance, and overlapping genetic/hormonal determinants. Across multiple cohorts, higher adiposity is associated with poorer glycemic control and increased risk of cardiovascular events and mortality, as well as higher risks of retinopathy, diabetic kidney disease, and neuropathy. Evidence to guide treatment in T1DM remains limited. Lifestyle approaches require individualization to minimize hypoglycemia and ketosis. RCTs of adjunct liraglutide demonstrate weight loss and insulin-sparing effects but signal dose-dependent risks of hypoglycemia and ketosis. Early RCTs with semaglutide and tirzepatide suggest substantial weight loss and improved glycemic metrics in selected settings, with vigilance for ketosis. Sodium-glucose cotransporter-2 inhibitors have shown modest HbA1c reductions with small but clinically meaningful weight loss in T1DM; however, they are associated with an increased risk of diabetic ketoacidosis. Bariatric surgery yields large weight loss but only modest glycemic benefit and carries risk of metabolic instability.

**Summary:**

Obesity is a common, clinically important comorbidity in T1DM, linked to poorer glycemic outcomes and complications. Longer-term, T1DM-specific RCTs are needed to guide weight management and define the benefit-risk of emerging therapies.

## Introduction

According to estimates from the International Diabetes Federation, in 2024 approximately 9.15 million people worldwide were living with type 1 diabetes mellitus (T1DM) [1]. Of this total, 19.8% were aged < 20 years, 68.6% were 20–59 years, and 11.8% were ≥ 60 years, underscoring that T1DM is predominantly an adult condition at the population level [1]. In the same year, an estimated 503,000 new T1DM diagnoses occurred across all ages [1].

In parallel with the increasing incidence of T1DM, the prevalence of obesity among people living with T1DM has also risen [2, 3]. This trend is clinically important because excess adiposity in T1DM is associated with more challenging glycemic management [4–6] and a higher risk of both microvascular [7–12] and macrovascular complications [13–18]. However, progress in addressing obesity in this population remains constrained by limited guideline-directed recommendations and the practical challenges of achieving meaningful weight loss while maintaining optimal glycemic control and minimizing hypoglycemia risk [2, 3].

In view of emerging evidence, particularly regarding the role of incretin-based therapies in the management of obesity in T1D, the aim of this narrative review was to summarize current evidence on the relationship between obesity and T1DM, the complications associated with excess adiposity in people with T1DM, and novel approaches to obesity management in this population. A literature search was performed using PubMed, Cochrane Library and Google Scholar (up to January 2026). Search terms combined “type 1 diabetes”/“T1DM” with “obesity”, “overweight”, “body mass index”, “adiposity”, “insulin resistance”, “cardiovascular”, “microvascular”, “retinopathy”, “nephropathy”, “neuropathy”, “MASLD/NAFLD”, “bone health/osteoporosis”, “weight management”, “diet”, “exercise”, “bariatric surgery”, and adjunct pharmacotherapies (e.g., “GLP-1 RA”, “semaglutide”, “liraglutide”, “tirzepatide”, “pramlintide”, “metformin”). Only English-language human studies were included.

## Relationship Between Obesity and T1DM

The global rise in obesity is well documented, and clear gradients have been described across age, sex, socioeconomic status, race and ethnicity, and lifestyle factors [19]. Comparable patterns in people with T1DM have been less well described, partly because weight loss was historically linked with poorly controlled T1DM during eras of suboptimal insulin therapy, making excess adiposity relatively uncommon.

In this review, overweight and obesity are defined using standard BMI categories (overweight: 25.0–29.9 kg/m²; obesity: ≥30.0 kg/m²) [19]. Because BMI does not capture fat distribution or body composition [19], we also consider obesity “phenotypes” where data are available, including central adiposity, assessed by waist circumference, waist-to-hip ratio, or waist-to-height ratio and body composition/ectopic fat, such as higher visceral adipose tissue or hepatic steatosis measured by dual-energy X-ray absorptiometry (DXA) or imaging.

### Prevalence of Obesity in T1DM

In adults with T1DM, the prevalence of overweight and obesity varies markedly across different regions of the world. The most robust contemporary estimates come from nationally representative surveys. In the pramlintide States (U.S.), a population-based analysis used pooled National Health Interview Survey (NHIS) data from 128,571 nonpregnant adults between 2016 and 2021 [20]. BMI was calculated from self-reported height and weight and categorized as normal weight (< 25 kg/m²), overweight (25 to < 30 kg/m²), or obesity (≥ 30 kg/m²). In this nationally representative sample, 64% of adults without diabetes (DM) and 62% of those with T1DM were living with overweight or obesity, compared with 86% of those with type 2 diabetes (T2DM), indicating that excess body weight is now almost as common in adults with T1DM as in the general U.S. population [20]. By contrast, estimates from registries and clinic-based cohorts are more heterogeneous and may be limited by incomplete BMI capture and selection bias. For example, in Mexico, a national T1DM registry recorded height and weight; among 965 participants (BMI available for 469), 42.4% were living with overweight and 8.1% with obesity [21]. These prevalences were lower than those reported in the Mexican general population, which has among the highest rates of obesity worldwide [22]. In Austria, a small clinic-based study in a small cohort of 186 adults with T1DM reported that the prevalence of overweight or obesity was comparable to that of the general population [23]. However, among participants aged 30–49 years, mean BMI was significantly higher in those with T1DM than in age-matched individuals without DM (26.7 kg/m² vs. 24.8 kg/m², *p* < 0.001) [23]. In Belgium, a large nationwide cohort (*n* = 89,834; ages 1–80 years) reported overweight/obesity prevalence similar to the general population and stable over the preceding decade [24].

Data in adolescents with T1DM are equally concerning, although estimates vary by study design and time period. In the T1DM Exchange Clinic Registry in the U.S., baseline data from 5,529 adolescents with T1DM (mean age 15.4) showed a substantial burden of excess adiposity, defined using BMI-based categories [25]. Overall, 22.9% were classified as living with overweight and 13.1% as living with obesity, corresponding to approximately 36% of the cohort. The prevalence of excess weight was particularly high among girls (40.8%) and adolescents of Hispanic/Latino ethnicity (46.1%) [25]. In children and adolescents, the population-based SEARCH for Diabetes in Youth study (2001–2004) in the U.S. compared 3,953 individuals with DM (aged 3–19 years) with 7,666 youth without DM from the National Health and Nutrition Examination Survey (NHANES) [26]. Weight status was assessed using BMI. Among youth with T2DM, 10.4% and 79.4% were living overweight and obesity, respectively; among those with T1DM, 22.1% and 12.6% were living with overweight and obesity (combined 34.7%), respectively. Youth with T1DM had a higher prevalence of overweight, but not of obesity, than youth without DM (22.1% vs. 16.1%; *p* < 0.05) [26].

In the SWEET registry, an international consortium of pediatric diabetes centers that prospectively collects clinical data from youth with T1DM worldwide, a cross-sectional analysis of 23,026 children and adolescents with T1DM (aged 2–18 years) showed that excess weight was common, with roughly one-third classified as overweight or living with obesity [27]. Using World Health Organization (WHO) BMI standard deviation score (BMI-SDS), the prevalence of overweight and obesity was 22.3% and 7.3% in males, and 27.2% and 6.8% in females, with BMI-SDS significantly higher in girls than in boys (*p* < 0.0001) [27].

Despite growing evidence that overweight and obesity are now common in people with T1DM across the life course, important limitations remain in the epidemiologic literature. Contemporary, population-level estimates are still sparse for several regions, particularly parts of Asia and Australia, and many available data derive from single-center cohorts or registries with incomplete anthropometric capture, limiting generalizability and cross-country comparisons. In addition, some widely cited pediatric estimates reflect earlier eras and may not fully capture current secular trends in adiposity. Heterogeneity in study design, BMI definitions, and age distributions further complicates interpretation. Although the drivers of geographic variation are incompletely understood and may relate to differences in diabetes care access and quality, these disparities should not foster complacency, as the increasing burden of excess adiposity is likely to adversely impact outcomes in T1DM. Moving forward, updated, standardized surveillance with consistent definitions and stratified reporting (by age, sex, and key subgroups) is needed to better quantify the global burden and inform targeted prevention and management strategies.

### Obesity as a Risk Factor of Developing T1DM

Obesity may increase the risk of developing T1DM, supporting a bidirectional relationship between the two conditions [28–30]. According to the “accelerator hypothesis”, genetic predisposition, insulin resistance and autoimmunity drive β-cell loss through apoptosis [28, 29]. In this framework, insulin resistance, which is strongly linked to excess adiposity, leads to β-cell failure and thereby contributes to the development of T1DM [28, 29].

The accelerator hypothesis was originally formulated by Wilking on the basis of a small cohort of 168 children and adolescents (aged 1.1–15.7 years) diagnosed with T1DM between 1980 and 2002 [31]. This study found an inverse correlation between age at T1DM diagnosis and BMI-SDS six months later (*r*=−0.30; *p* < 0.001). This suggests that individuals with a higher BMI tend to develop or be diagnosed with T1DM at a younger age compared to those with a lower BMI [31].

Epidemiological evidence has provided broader support for this concept. A meta-analysis of 4 studies that treated childhood obesity as a categorical exposure reported a pooled odds ratio (OR) of 2.03 [95% Confidence Intervals (CI): 1.46–2.80)] for subsequent T1DM [32]. Using a life course Mendelian randomization approach, recent genetic evidence further supports a causal role of higher childhood body size in increasing the risk of T1DM [33]. In univariable analyses, each increment in childhood body size category was associated with approximately a twofold higher odd of T1DM [OR (95% CI): 2.05 (1.20–3.50)], and this effect persisted after adjustment for birth and adult body size in multivariable models [OR (95% CI): 2.32 (1.21–4.42)]. These findings were validated in a large T1DM meta-analysis including 15,573 cases and 158,408 controls [OR (95% CI): 1.21–3.12)], reinforcing the concept that greater childhood adiposity directly increases the risk of subsequent T1DM [33].

Apart from the child’s body weight, familial factors, particularly maternal body weight, may also influence T1DM risk. A meta-analysis of 21 studies reported that, compared with mothers with normal weight, maternal overweight and maternal obesity were associated with a higher risk of childhood-onset T1DM [relative risk (RR) (95% CI): 1.09 (1.03–1.15) and RR (95% CI): 1.25 (1.16–1.34), respectively] [34]. In addition, paternal obesity has also been linked to childhood-onset T1DM. In a pooled analysis of 132,331 children, both maternal pre-pregnancy obesity [adjusted hazard ratio (HR) (95% CI): 1.41 (1.06–1.89) and paternal obesity [adjusted HR (95% CI): 1.51 (1.11–2.04)] were associated with incident childhood-onset T1DM, with broadly similar estimates after mutual adjustment [35].

In summary, available data suggest that greater adiposity, particularly in childhood, may increase T1DM risk and/or accelerate progression to clinical disease, although certainty is limited by heterogeneous study designs and potential confounding, and it remains unclear whether adiposity primarily influences disease initiation versus acceleration. Mechanistically, elevated circulating fatty acids and glucose may impair β-cell function and survival, potentially increasing susceptibility to immune-mediated destruction [36]. Robust prospective cohorts are therefore needed in children and adults living with overweight or obesity who are at increased T1DM risk (e.g., positive islet autoantibodies or family history) to clarify causality and identify susceptible subgroups. These studies should use adiposity measures beyond BMI (e.g., waist circumference, waist-to-hip ratio, and body fat percentage), and interventional work is required to determine whether effective weight control can prevent or delay progression to T1DM and help mitigate the ongoing rise in T1DM incidence.

### Factors Contributing to Weight Gain in T1DM

The contemporary obesogenic environment, characterised by widespread availability of energy-dense foods and increasingly sedentary lifestyles, has been a major driver of the obesity epidemic. Nevertheless, the factors contributing to weight gain in people with T1DM have received comparatively less attention [3].

A key contributor is intensive insulin therapy, the cornerstone of T1DM management, which can paradoxically promote weight gain. Although the mechanisms underlying insulin-associated weight gain are not fully elucidated, several explanations have been proposed [2, 3]. First, improved glycemic control reduces plasma glucose below the renal threshold for excretion, leading to decreased glycosuria and greater retention of calories [2]. Second, exogenous insulin is delivered peripherally rather than through the portal circulation, bypassing first-pass hepatic extraction and potentially resulting in relative peripheral hyperinsulinemia that favors lipogenesis and fat accumulation. Additional mechanisms have also been suggested, including changes in the growth hormone/IGF-1 axis, which plays an important role in regulating body composition through the balance of anabolic and catabolic processes [2, 3].

The extent to which the mode of insulin delivery influences weight trajectory remains debated. Although continuous subcutaneous insulin infusion (CSII) has been suggested to promote greater weight gain than multiple daily injections (MDI), this hypothesis is not supported by high-quality prospective randomized controlled trial (RCT) evidence [2]. In a decade-long retrospective comparison of CSII versus MDI, weight gain did not differ between groups; however, CSII was associated with greater improvements in glycemic control and lower daily insulin requirements [37]. Notably, in the Diabetes Control and Complications Trial (DCCT), weight gain was also observed in the intensively treated arm irrespective of the insulin replacement modality, underscoring that intensification itself, rather than delivery method, may be a key determinant [38].

Furthermore, the prevalent notion that T1DM solely reflects insulin deficiency is inaccurate, as insulin resistance is also an important component of the disease [39]. Obesity promotes insulin resistance and increases insulin requirements, creating a self-reinforcing cycle in which escalating insulin doses may further contribute to weight gain [3]. Insulin resistance can be present in people with T1DM, may be detectable early, and can progress over time in a tissue-specific manner [39]. Euglycemic-hyperinsulinemic clamp studies support distinct defects across key insulin-sensitive tissues [39]. Hepatic insulin resistance may be partly related to nonphysiological portal insulin gradients with subcutaneous insulin and may be compounded by relatively elevated glucagon levels and increased nonesterified fatty acid (NEFA) flux. Skeletal muscle insulin resistance is a consistent finding in T1DM and limits glucose disposal and glycogen synthesis [39]. Adipose tissue insulin resistance impairs suppression of lipolysis, further increasing circulating NEFAs [39]. Vascular dysfunction may additionally restrict insulin delivery through reduced capillary recruitment.

Lifestyle-related factors and nonphysiological peripheral insulin delivery may contribute to insulin resistance in T1DM by promoting glucotoxicity, lipotoxicity, iatrogenic hyperinsulinemia, mitochondrial dysfunction, oxidative stress, and inflammation. However, these mechanisms do not fully explain insulin resistance in T1DM, suggesting that additional disease-specific determinants are involved [39]. Emerging evidence also supports immunometabolic heterogeneity, with a subset of T1DM (“endotype 2”) exhibiting a more pronounced insulin-resistant phenotype [39]. Accordingly, targeting insulin resistance in T1DM may help attenuate cardiometabolic risk and long-term complications, and should be considered an additional therapeutic objective alongside weight management, as discussed in the section “Pharmacological Management of Obesity.”

**Fig. 1:**
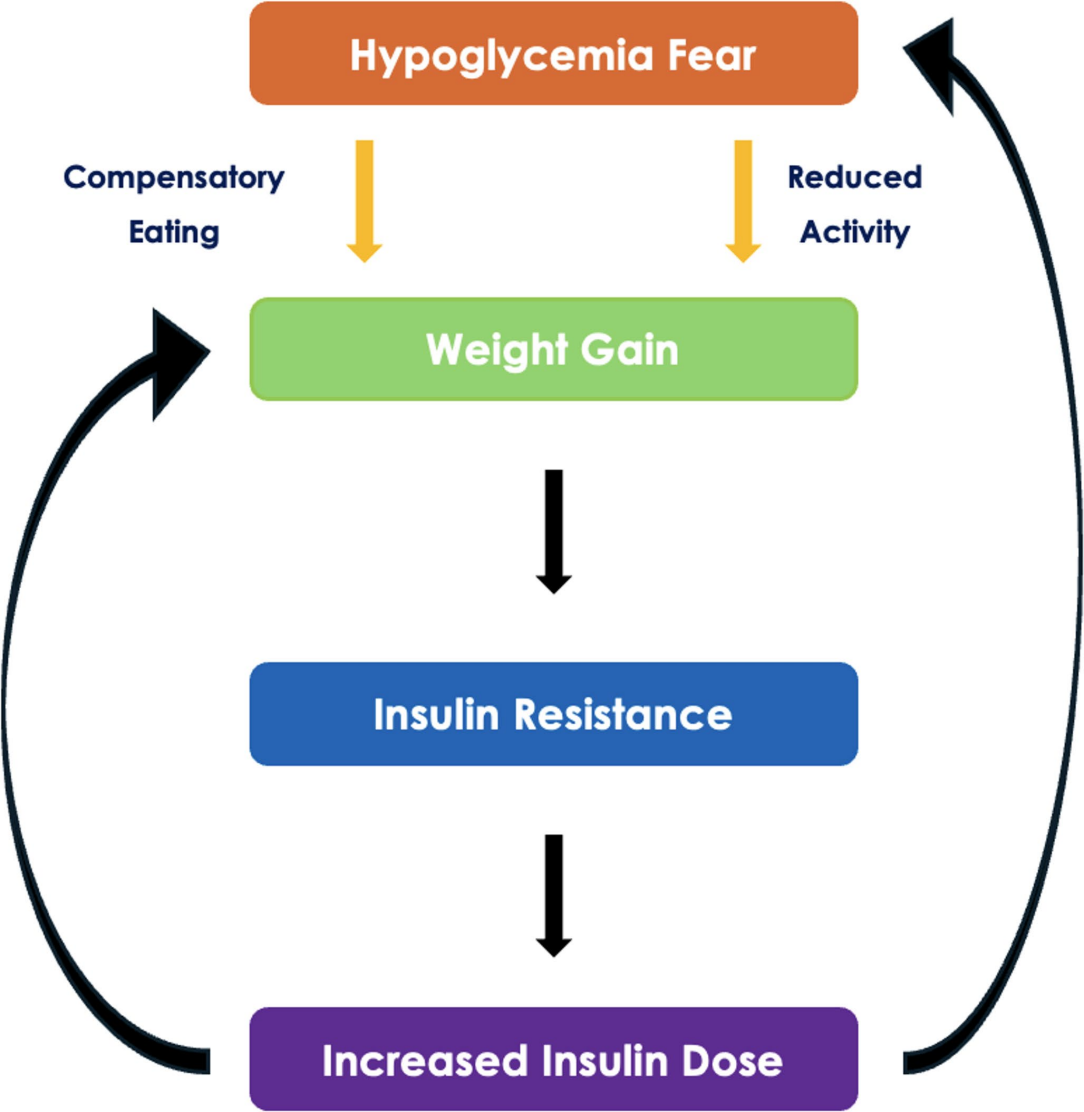
Hypoglycemia-insulin dose-eating behavior feedback loop driving weight gain in T1DM

Another driver of weight gain with intensive insulin therapy is the heightened risk of hypoglycemia [2]. To prevent or treat low glucose levels, people with T1DM often increase carbohydrate intake, including “defensive” snacking (e.g., before exercise) and compensatory eating during hypoglycemic episodes, which can contribute to positive energy balance and weight gain [2, 3]. Fear of hypoglycemia may also restrict participation in physical activity, as mentioned above, contributing to lower rates of meeting recommended exercise targets (Fig. 1).

Beyond behavioral and treatment-related drivers, familial and genetic factors are also likely to contribute to excess weight in people with T1DM. Consistent with this, as mentioned above, children of parents living with obesity appear to have a higher risk of developing childhood-onset T1DM [34]. At the genetic level, susceptibility may partially overlap between obesity and T1DM [40]. Variants in *FTO*, a gene involved in appetite regulation and eating behavior, have been associated with a higher likelihood of overweight and poorer metabolic control in children with T1DM [41]. In addition, in a large T1DM cohort, *TCF7L2* variants, classically linked to T2DM, were associated with a milder immunologic phenotype and greater residual endogenous insulin secretion [42].

Furthermore, hormonal abnormalities in T1DM, including impaired secretion and/or action of insulin, glucagon, amylin, leptin, and other enteroendocrine hormones, can disrupt appetite regulation and energy homeostasis, thereby impairing the normal control of food intake [43].

Overall, weight gain in T1DM is multifactorial, arising from the interaction of an obesogenic environment with treatment-related (intensive insulin therapy, hypoglycemia avoidance and compensatory eating, and behavioral adaptations), metabolic (insulin resistance and hyperinsulinemia), and biological determinants (hormonal dysregulation and genetic susceptibility). The strength of evidence is greatest for insulin intensification and hypoglycemia-related behaviors, whereas the relative contribution of delivery modality and specific genetic/hormonal pathways is less certain and likely varies across subgroups and life stages. Clinically, prevention requires a proactive, individualized approach that integrates optimized insulin strategies (including technology where appropriate), structured hypoglycemia risk mitigation, and targeted lifestyle and behavioral support, while recognizing insulin resistance as an additional therapeutic target alongside weight management.

### Sarcopenic Obesity in T1DM

Sarcopenic obesity, characterized by reduced skeletal muscle mass and function in the presence of excess adiposity, has been extensively studied in people with T2DM. Nevertheless, data in T1DM remain limited [44]. In T1DM, the available literature has focused predominantly on sarcopenia rather than sarcopenic obesity per se [45].

In this context, unfavorable body composition may reflect chronic hyperglycemia, insulin deficiency, and diabetes-related complications, which can accelerate muscle loss and promote fat accumulation. Observational studies suggest that older age, longer diabetes duration, poorer glycemic control, and microvascular complications, particularly diabetic kidney disease, are associated with higher sarcopenia risk in people with T1DM, although findings are not fully consistent across cohorts [45]. Mechanistically, chronic inflammation, insulin resistance, and hormonal perturbations may further contribute to muscle catabolism and adipose gain [44, 45]. Clinically, sarcopenic obesity may increase frailty, functional impairment, and cardiometabolic and renal risk, highlighting the importance of assessing body composition (e.g., DXA or bioimpedance) rather than relying on BMI alone. Well-designed, T1DM-specific longitudinal studies are needed to define the prevalence, determinants, and prognostic impact of sarcopenic obesity and to identify effective prevention and treatment strategies.

## Complications of Obesity in T1DM

### Impact of Obesity on Glycemic Control

Obesity in people with T1DM adds a substantial layer of complexity. In this section, we summarize the evidence on how excess adiposity affects glycemic control in children, adolescents and adults with T1DM.

In a large analysis of pediatric T1DM (median age 12.6 years), data from two large clinical registries in Germany/Austria and the U.S. (*n* = 32,936) showed a consistent adverse association between adiposity and glycemic control [5]. Across both cohorts, higher BMI z-scores were significantly associated with higher HbA1c levels, indicating poorer glycemic control, and with a greater frequency of severe hypoglycemia episodes [5]. This paradox has been partly attributed to excess body weight, which may contribute to impaired hypoglycemia awareness; however, it has not been replicated in subsequent studies, and the underlying mechanisms remain unclear [4]. Similarly, in adults with T1DM, a multicenter observational study including 719 individuals (mean age 41.5 ± 13.9 years) showed that participants with higher BMI had higher HbA1c levels [6].

On the other hand, a smaller cross-sectional continuous glucose monitor (CGM)-based study in 73 adults with T1DM (mean age 39 years) did not observe similar associations [46]. The proportion achieving optimal glycemic targets was similar across normal-weight, overweight, and individuals living with obesity. BMI was paradoxically positively associated with better glycemic control; however this finding likely reflects the limitations of using BMI alone to assess obesity, rather than evidence of a true “obesity paradox” in T1DM [46]. The analysis was based on CGM-derived metrics recorded over the preceding 14 days, which may be too short to fully capture typical glycemic patterns. Moreover, the small sample size limits the robustness of these findings, and larger, longer CGM-based studies are needed to determine whether this pattern truly holds.

Finally, weight gain can also negatively affect adherence to insulin therapy. Lack of adherence is common in people with T1DM, with estimates ranging from 23% to 77% [47], and may be more pronounced in those who fear gaining weight. In some individuals, this leads to intentional underdosing or omission of insulin to promote weight loss, thereby increasing the risk of diabetic ketoacidosis (DKA) as well as long-term diabetes complications. An earlier U.S. study from 1994 involving 341 girls and women with T1DM (aged 13–60 years) reported that 31% deliberately omitted insulin, with 9% doing so frequently, and approximately half of those who omitted insulin indicating that their primary motivation was weight control [48].

To sum up, the preponderance of evidence, largely from observational cohorts, supports an adverse association between excess adiposity and glycemic control in T1DM, particularly in pediatric populations where higher BMI z-scores correlate with higher HbA1c. However, findings are not entirely consistent, as smaller CGM-based studies have reported null or paradoxical associations, likely reflecting limited sample size, short CGM observation windows, and the constraints of BMI as a sole adiposity metric. Notably, CGM-derived indices provide complementary insight into glycemic quality beyond HbA1c and have been consistently linked to microvascular risk, supporting their use as additional endpoints in this context [49]. Clinically, obesity may also worsen glycemic outcomes indirectly through hypoglycemia-related behaviors and weight concerns that undermine insulin adherence, increasing the risk of DKA. Future studies should leverage longer-duration CGM metrics and more precise measures of adiposity and fat distribution in longitudinal and interventional designs to clarify causality and identify actionable strategies to improve glycemic control in people with T1DM living with overweight or obesity.

### Impact of Obesity on Cardiovascular Disease and Mortality

**Fig. 2:**
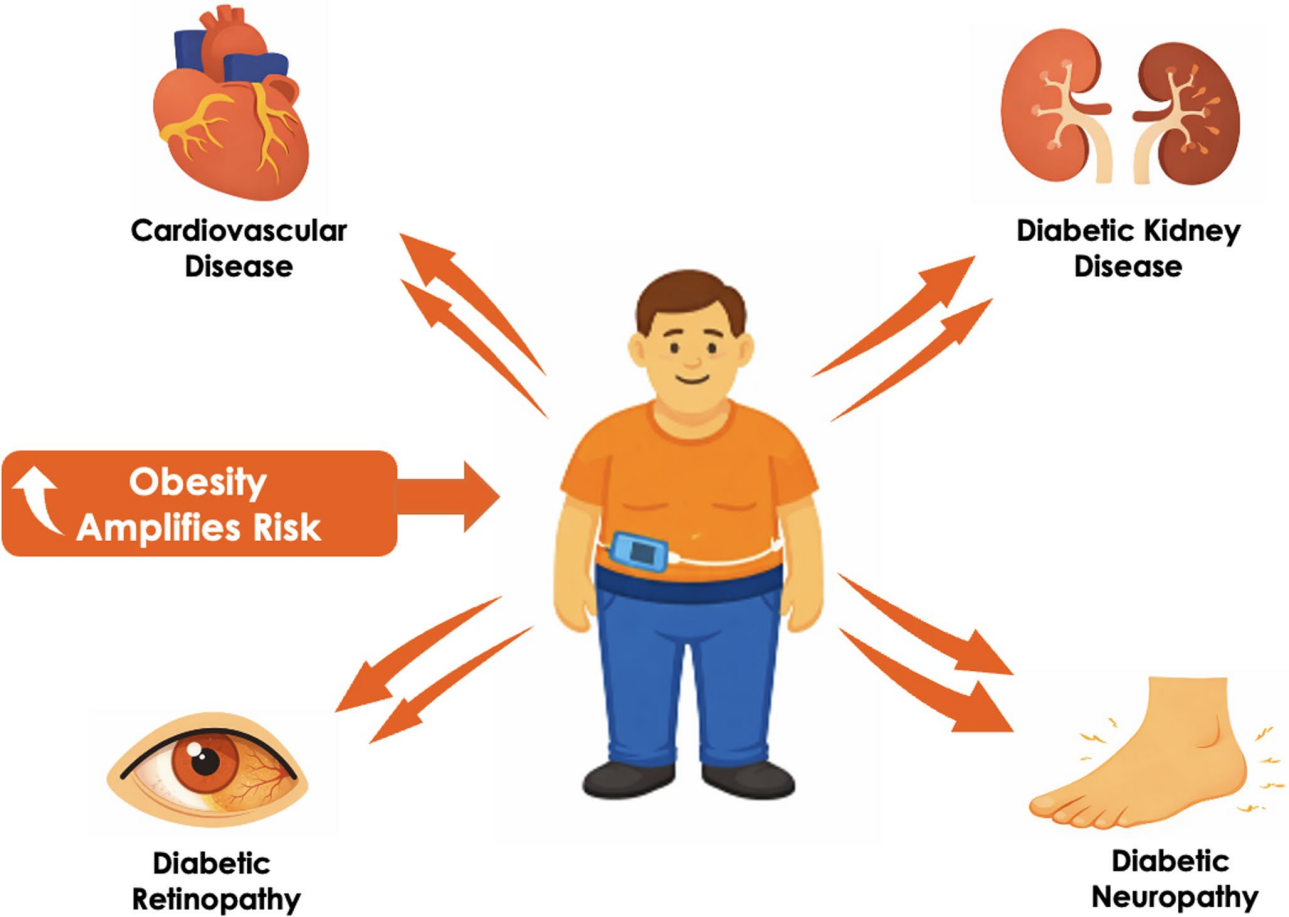
Obesity amplifies diabetes-related complication risk in T1DM

Despite substantial improvements in DM management, individuals with T1DM still experience a markedly reduced life expectancy, estimated to be around 13 years shorter than that of the general population [13]. This excess mortality is driven predominantly by CV disease, and people with T1DM remain at consistently higher CV risk across the life course compared with individuals without DM (Fig. 2).

In a nationwide analysis from the Swedish National Diabetes Register, 10,184 individuals with adult-onset T1DM and 375,523 individuals with T2DM diagnosed between 2001 and 2020 were compared with 509,172 population controls from the Total Population Register and followed until 2022 [14]. In people with adult-onset T1DM, smoking, BMI, and HbA1c emerged as the principal contributors to adverse outcomes, collectively accounting for approximately 40% of CV events and 20% of deaths, underscoring the central role of modifiable cardiometabolic risk factors in shaping long-term prognosis [14].

Similarly, another nationwide analysis from the Swedish National Diabetes Registry, 26,125 adults with T1DM (mean age 33.3 years) were followed between 1998 and 2012. A BMI > 25 kg/m^2^ was associated with an increase in risk of mortality, major cardiovascular (CV) disease, and heart failure [15]. Notably, these associations were observed in men but were not observed in women.

In the Finnish Diabetic Nephropathy Study, 4,668 adults with T1DM were included in an analysis assessing the impact of general and central obesity on the risk of heart failure [16]. General obesity was defined as BMI ≥ 30 kg/m², whereas central obesity was defined as a waist-to-height ratio ≥ 0.5. Over a median follow-up of 16.4 years, central adiposity and general adiposity, were both associated with heart failure risk. However, the association was stronger for central adiposity [HR (95% CI): 1.51 (1.26–1.81)] than for general adiposity [HR (95% CI): 1.05 (1.01–1.08)] [16].

In a large, contemporary U.S. cohort of adults living with T1DM, CV risk factors were prospectively evaluated in 8,727 participants (median age 33 years) [17]. Over a mean follow-up of 4.6 years, 325 individuals (3.7%) developed CV disease. Higher BMI was associated with an increased risk of CV disease.

In a retrospective analysis of 2,181 adults with T1DM enrolled in a nationwide Japanese registry between 2016 and 2023, participants were categorized by overweight status (overweight vs. not overweight) [18]. Coronary artery disease was more frequent in the overweight group than in the non-overweight group (8.9% vs. 3.4%; *p* < 0.001), and overweight remained independently associated with coronary artery disease [adjusted OR (95% CI): 2.41 (1.15–5.06)]. Interestingly, no significant associations were observed between overweight and overall CV disease or peripheral arterial disease.

Collectively, the available evidence, predominantly from large, observational cohorts, supports excess adiposity as a clinically relevant, modifiable contributor to CV disease, heart failure, and mortality in T1DM. Associations are generally consistent across settings, with signals that central adiposity may convey greater risk than BMI alone, and that effect estimates may differ by sex and population, underscoring heterogeneity in susceptibility and the importance of subgroup interpretation. While causality cannot be fully inferred from observational designs, the convergence of findings across multiple registries strengthens confidence that weight and adiposity distribution should be routinely addressed alongside traditional risk factors (e.g., HbA1c, smoking, blood pressure, and lipids) to reduce long-term CV burden in people with T1DM.

### Impact of Obesity on Microvascular Complications

Obesity may also influence the risk and progression of microvascular complications in T1DM, potentially exacerbating diabetic retinopathy, diabetic kidney disease, and neuropathy beyond the effects of hyperglycemia alone. The contribution of obesity to these complications is further underscored by evidence that traditional “diabetic” complications, such as neuropathy, can also occur in people living with obesity in the absence of diabetes [50]. Likewise, obesity per se can increase intraglomerular pressure and promote the development and progression of chronic kidney disease (CKD) [51]. A summary of observational studies examining the relationship between body weight and microvascular complications in T1DM is provided in Table 1.

**Table 1:** Association of body weight with microvascular complications in T1DM: summary of observational studies

Study	Design & Setting	Population	Key Findings
Retinopathy			
Nystrom 2024 [7]	Population-based cohort	21,575 individuals with T1DM, age 0–39 years	Overweight/obesity was associated with an increased risk of retinopathy, including among individuals whose BMI subsequently returned to the normal range [HR (95% CI): 1.30 (1.12–1.51)], with estimates comparable to those with persistent overweight/obesity [HR (95% CI): 1.31 (1.20–1.43)] Following multivariable adjustment, the association remained significant only in those with persistent overweight/obesity [HR (95% CI): 1.14 (1.04–1.24)]
Price 2014 [9]	Prospective hospital-based cohort (specialist clinic database)	501 adults with T1DM	Obesity was a risk factor for retinopathy, despite similar HbA1c across BMI categories
Laginhas 2019 [10]	Retrospective chart review (tertiary centre)	Early-onset < 18 years T1DM (*n* = 140) vs. late-onset ≥ 18 years (*n* = 169) T1DM	Higher BMI was significantly associated with proliferative diabetic retinopathy in the early-onset group, but not in the late-onset group
Diabetic kidney disease			
Habte-Asres 2019 [11]	Population-based retrospective cohort	1,106 adults with T1DM	Higher BMI was independently associated with reduced eGFR
Neuropathy			
Tesfaye 2005 [8]	Multicentre European prospective cohort	1,172 adults with T1DM	Higher BMI was independently associated with incident neuropathy (peripheral and cardiac autonomic neuropathy) after adjustment for other risk factors

*T1DM* type 1 diabetes, *HR* hazard ratio, *BMI* body mass index, *eGFR* estimated glomerular filtration rate

In a population-based cohort study from the Swedish National Diabetes Registry, 21,575 individuals with T1DM aged 0–39 years were followed between 1998 and 2017 [7]. In models adjusted only for calendar year, overweight/obesity was associated with a higher subsequent incidence of retinopathy; this association was observed both among individuals who had overweight/obesity at any time point, including those whose BMI later returned to the normal range [HR (95% CI): 1.30 (1.12–1.51)], and among those with persistent overweight/obesity [HR (95% CI): 1.31 (1.20–1.43)]. After multivariable adjustment for potential confounders, the association with retinopathy remained statistically significant only among individuals with persistent overweight/obesity [HR (95% CI): 1.14 (1.04–1.24)] [7].

In an Australian hospital-based cohort, 501 adults with T1DM attending a specialist clinic were identified, and clinical and biochemical data were prospectively collected between 1998 and 2011 using a dedicated patient management database [9]. In both men and women, obesity (BMI ≥ 30 kg/m²) was a predominant risk factor for retinopathy, despite comparable HbA1c levels across BMI categories.

In a retrospective study assessing risk factors for diabetic retinopathy in T1DM, medical records were reviewed for all individuals with T1DM from a tertiary center [10]. An analysis was performed comparing patients diagnosed before 18 years of age (early-onset group, *n* = 140) and those diagnosed at or after 18 years of age (late-onset group, *n* = 169). Interestingly, higher BMI was significantly associated with proliferative diabetic retinopathy in the early-onset group, whereas no such association was observed in the late-onset group [10].

Consistent with these ocular findings, obesity also appears to adversely affect renal outcomes in T1DM. In a population-based retrospective cohort study from UK electronic health records were used to examine CKD risk in 1,106 adults with T1DM. Over follow-up, 38.5% developed reduced estimated glomerular filtration rate (eGFR), 47.2% had albuminuria, and 23.8% met both criteria for CKD [11]. Higher BMI was independently associated with reduced eGFR, highlighting excess adiposity as an important contributor to renal risk in T1DM [11].

A similar pattern is observed for neuropathic complications. In the European Diabetes (EURODIAB) Prospective Complications Study, risk factors for incident neuropathy (peripheral and cardiac autonomic neuropathy) were evaluated in 1,172 adults with T1DM from 31 centers across Europe [8]. Neuropathy was assessed at baseline (1989–1991) and again at follow-up (1997–1999), over a mean follow-up of 7.3 years. By follow-up, neuropathy had developed in 276 of the 1,172 participants who were free of neuropathy at baseline (23.5%). After adjustment for other risk factors and diabetic complications, higher BMI remained independently associated with the incidence of neuropathy [8].

In a systematic review, higher waist-to-height ratio, a marker of central adiposity, was consistently associated with the presence or development of both peripheral neuropathy and cardiac autonomic neuropathy in children, adolescents, and young adults with T1DM [12]. Building on these observations, a recent DXA-based study that included both T1DM and T2DM reported that visceral adipose tissue mass and markers of central/ectopic adiposity (including trunk-to-leg fat ratio) were more strongly associated with CAN in T1DM than in T2DM [52]. This supports a role for central fat distribution as a contributor to dysautonomia risk in T1DM independently of BMI.

Available evidence, almost exclusively observational, suggests that excess adiposity contributes meaningfully to microvascular risk in T1DM, with generally consistent signals for retinopathy, diabetic kidney disease, and neuropathy even after adjustment for glycemic control and other risk factors. However, effect sizes vary across cohorts and subgroups (e.g., early- vs. late-onset T1DM), and residual confounding and differences in outcome definitions limit causal inference. In addition, adiposity may reflect both lifestyle/socioeconomic factors and mediating pathways (e.g., insulin resistance leading to higher insulin requirements). Nvertheless, the relative contribution of these mechanisms remains uncertain, as they are not uniformly measured or captured across studies.

Importantly, emerging data indicate that central/ectopic adiposity (e.g., waist-to-height ratio, visceral fat, trunk-to-leg ratio) may better capture risk than BMI alone, particularly for neuropathic outcomes such as cardiac autonomic neuropathy. Clinically, these findings support incorporating weight and fat-distribution assessment into routine complication risk stratification and prioritizing integrated interventions that address adiposity alongside established microvascular risk factors in T1DM.

### Impact of Obesity on MASLD

Metabolic dysfunction-associated steatotic liver disease (MASLD) is defined by the coexistence of hepatic steatosis with at least one of the five typical traits of the metabolic syndrome, in the absence of clinically significant alcohol consumption and other secondary causes of steatosis [53]. As overweight and have become increasingly prevalent in people with T1DM, MASLD is being recognized with meaningful frequency in this population.

In a recent systematic review and meta-analysis including 23 studies and 13,006 adults with T1DM, MASLD was found to be relatively common, with a pooled prevalence of 22.2% (95% CI: 15.6–30.7) [54]. Among people with T1DM and MASLD, the pooled prevalence of significant fibrosis (≥ F2) was 13.3% (95% CI: 11.2–15.7) and advanced fibrosis (≥ F3) was 5.1% (95% CI 3.8–6.9). Individuals with T1DM and MASLD were more likely to be older, male, living with overweight, have longer diabetes duration, higher daily insulin requirements, and greater metabolic dysfunction, and they also exhibited a higher risk of microvascular complications compared with those with T1DM without MASLD [54]. Reflecting the emerging relevance of MASLD in T1DM, the American Diabetes Association (ADA) recommends screening for fibrosis in people with T1DM when risk factors for MASLD are present, particularly obesity, elevated aminotransferases, or steatosis as an incidental imaging finding [55].

For context, MASLD appears more prevalent in T2DM, with a meta-analysis of 156 studies (1.8 million individuals) reporting a global prevalence of 65% (95% CI 61.8–68.2) [56], whereas estimates in the general adult population are commonly in the range of 25–35%, depending on the assessment method and population characteristics [53, 57, 58].

Notably, imaging-based studies provide additional insight regarding hepatic fat burden in T1DM. In a population-based Maastricht Study, liver fat content was assessed using liver MRI, the gold-standard non-invasive imaging modality for the quantification of hepatic steatosis and fibrosis [59]. The study included 29 individuals with T1DM, 58 with T2DM, and 58 with normal glucose metabolism, matched for BMI, age, sex, and educational level. Individuals with T1DM had significantly lower liver fat content than those with T2DM and levels comparable to participants without DM. These differences were independent of obesity, lifestyle factors, dietary intake, and CV risk factors, but were attenuated after adjustment for insulin sensitivity and insulin treatment, suggesting that insulin resistance and/or insulin therapy may partly account for between-group differences [59]. Consistently, a cross-sectional study using liver MR and MR elastography evaluated hepatic steatosis and fibrosis-related stiffness in 31 people with Latent Autoimmune Diabetes in Adults **(**LADA), matched to 31 with T2DM (sex, BMI, and DM duration) and 31 with T1DM (sex, BMI, and age) [60]. Liver fat was quantified using proton density fat fraction (PDFF), while liver stiffness was measured by MR elastography; visceral adipose tissue was additionally assessed by DXA. Steatosis (PDFF > 5.5%) was markedly more prevalent in T2DM (54.8%) than in LADA (3.2%) and was absent in T1DM (0%) (*p* < 0.001), whereas liver stiffness did not differ across groups. Visceral adipose tissue correlated with PDFF in the overall cohort, while BMI tracked liver fat content and liver stiffness only in T2DM [60]. Collectively, these findings suggest that hepatic fat accumulation and determinants of liver stiffness vary across autoimmune and non-autoimmune diabetes phenotypes, likely reflecting differences in body fat distribution despite similar BMI.

Overall, the available evidence suggests that hepatic steatosis and MASLD are clinically relevant, yet potentially under-recognized, comorbidities in T1DM that are associated with adiposity and broader metabolic risk profiles, whereas advanced fibrosis appears to occur in a smaller subset. At the same time, imaging-based studies indicate that, after careful matching for adiposity and other key confounders, hepatic fat content in people with T1DM may be comparable to that of individuals without DM and lower than that observed in T2DM. Collectively, these data point to heterogeneity in MASLD risk within T1DM and highlight the need for larger, well-designed longitudinal studies with standardized imaging-based phenotyping to better define disease burden, determinants, and long-term hepatic and cardiometabolic outcomes.

### Impact of Obesity on Bone Health

T1DM is associated with an increased risk of osteoporosis and fractures [61, 62]. Compared with the general population, fracture risk in people with T1DM is approximately 4.4-fold higher for hip fractures, 1.83-fold higher for upper-limb fractures, and 2-fold higher for ankle fractures [63]. Accordingly, the ADA recommends monitoring bone mineral density using DXA in older adults with DM (≥ 65 years) and in younger individuals with DM who have multiple risk factors, typically every 2–3 years [62].

In the general population, low BMI is associated with a higher risk of osteoporosis, likely reflecting reduced mechanical loading, whereas excess adiposity is often associated with higher areal bone mineral density. However, the relationship between obesity and bone health appears more complex in T1DM [61]. In an 18-month prospective study of 136 youth with T1DM (8–17 years), with DXA assessments at baseline, 12, and 18 months, greater adiposity and more central fat distribution were associated with less favorable bone outcomes [64]. Higher C-reactive protein levels were inversely associated with total-body, pelvic, and leg bone mineral density, while higher percent body fat was inversely associated with total-body and pelvic bone mineral density; percent trunk fat was inversely associated with total-body bone mineral density. Collectively, these findings suggest that adiposity (including central adiposity) and inflammation are linked to lower bone mineral density in youth with T1DM and may contribute to altered osteometabolic pathways [64].

In addition, osteocalcin is a vitamin K-dependent, osteoblast-derived protein and a key biomarker of bone formation. In a cohort of 62 people with T1DM (mean age 51 years), higher osteocalcin levels were inversely associated with an upper-body fat deposition index, defined as the ratio of upper-body fat mass (head, arms, trunk) to lower-body fat mass (hips and legs) (adjusted β −0.484; *p* = 0.001) [65].

Overall, both overall adiposity and adipose distribution may influence bone health and related osteometabolic pathways in T1DM. However, further studies are needed to clarify the role of adiposity in bone health in people with T1DM.

## Management of Obesity in T1DM

**Fig. 3:**
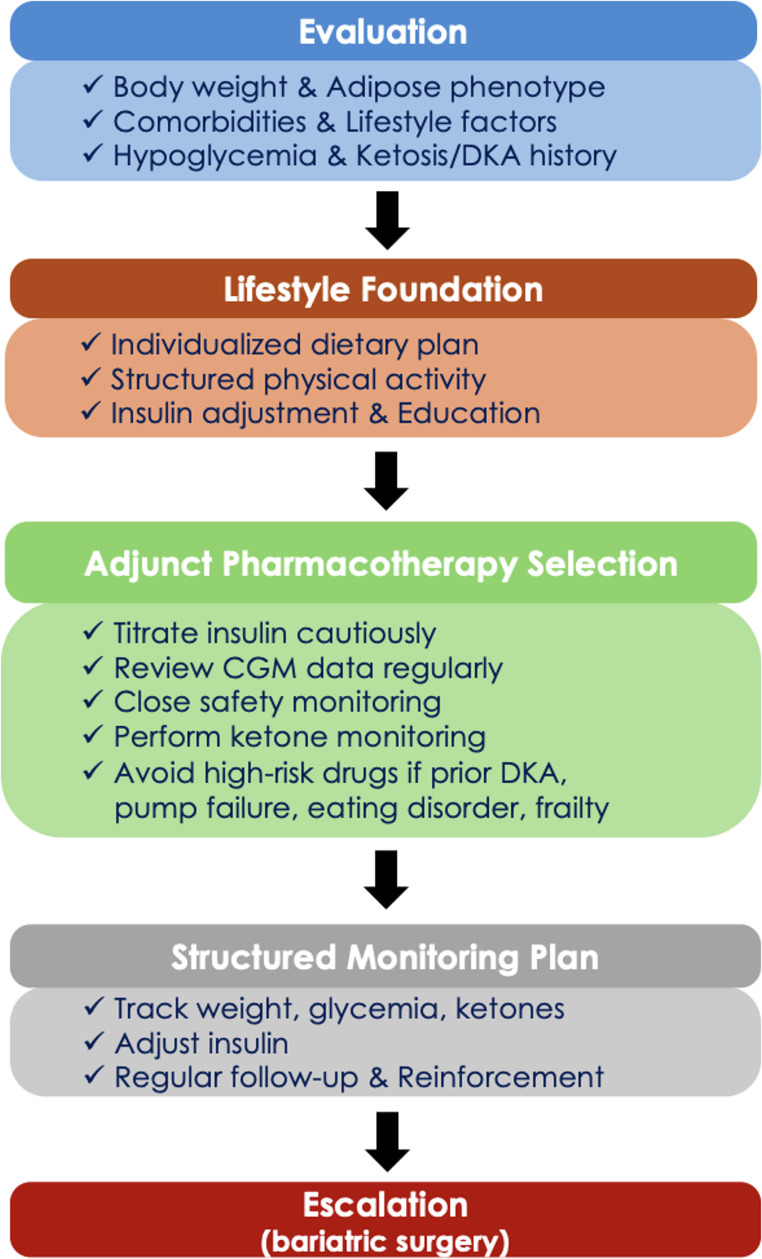
Proposed management algorithm for obesity in T1DM

As T2DM accounts for most of DM cases and obesity has traditionally been regarded as a hallmark comorbidity of this condition, most evidence-based weight management strategies in DM have been developed, tested, and implemented primarily in people with T2DM. Consequently, data specifically addressing the management of obesity in T1DM remain scarce. The following section outlines the available approaches to obesity treatment in T1DM. A proposed management algorithm for obesity in T1DM is presented in Fig. 3.

### Lifestyle and Behavioral Modifications

Management of obesity requires a multidisciplinary strategy that incorporates lifestyle and behavioral interventions, including dietary modification and increased physical activity.

Dietary strategies for weight management and metabolic control have been developed and evaluated predominantly in the context of T2DM. Well-designed RCTs examining dietary approaches, such as low carbohydrate or ketogenic diets, that simultaneously optimize weight management and glycemic control in T1DM are limited, and important safety questions remain incompletely addressed [66]. Low-carbohydrate (including ketogenic) diets are associated with several concerns, particularly an increased risk of hypoglycemia [66]. For example, a small observational study of 11 adults with T1DM showed that a ketogenic diet for a mean ± standard deviation of 2.6 ± 3.3 years, was associated with optimal HbA1c levels (5.3 ± 0.4%) and minimal glycemic variability, but also with dyslipidemia and a high frequency of hypoglycemic episodes [67]. In addition, such diets may deplete glycogen stores and may thereby predispose to DKA in people with T1DM [66].

The evidence available to date is inconsistent and sometimes conflicting. A 3-month pilot RCT in adults aged 19–30 years with T1DM and a BMI 27–40 kg/m^2^ (*n* = 38) compared the effect of three dietary patterns: a hypocaloric low carbohydrate diet, a hypocaloric Look AHEAD diet (i.e., moderate low fat), and a Mediterranean diet (not calory restricted), on body weight and glycemic indices [68]. The results showed no clear advantage of caloric restriction for combined weight (average 2.7 kg) and glucose outcomes, implying that patient preference and sustained adherence may be the principal drivers of dietary effectiveness. Furthermore, a review of eight studies examining low-carbohydrate diets in T1DM found inconsistent effects on BMI and total daily insulin dose (TDD) [69]. The small sample sizes across these studies prevented a meta-analytic assessment [69].

These findings indicate that, in people with T1DM, weight management is most appropriately pursued through individualized dietary plans that emphasize sustainable caloric reduction while ensuring safety, particularly regarding the risks of both hypoglycemia and hyperglycemia. Well-designed RCTs are required to define the optimal nutrition-based strategies for individuals with T1DM living with obesity.

Similar to dietary strategies, data regarding weight loss through exercise in individuals with T1DM are limited. A comprehensive summary in individuals with T1DM has been thoroughly described [70, 71]. Briefly, a central challenge is that exercise in T1DM is associated with an increased risk of hypoglycemia, which constitutes one of the major barriers to regular physical activity [70, 71]. A multicenter pilot RCT (ISRCTN61403534) is currently assessing the effectiveness and cost-effectiveness of a structured education program (The EXercise in people with Type One Diabetes, EXTOD) for people with T1DM [72]. Furthermore, advances in hybrid closed-loop systems could also enhance safety during physical activity. These systems improve insulin delivery and glucose level alignment and potentially reduce the risk of hypoglycemia during exercise.

To sum up, the evidence base for lifestyle-based weight management in T1DM remains limited and heterogeneous, with few adequately powered RCTs and most data derived from small observational studies or short-duration pilots. The modest weight changes reported in available trials (e.g., 2–3 kg over 3 months) are clinically meaningful for some individuals, but do not yet establish superiority of any single dietary pattern over another; sustained adherence and safety appear to be the dominant determinants of benefit. Applicability also varies across key T1DM subgroups: low-carbohydrate/ketogenic approaches may be higher risk in individuals with prior DKA, those prone to ketosis, those using large insulin dose reductions, or those with eating disorder risk/insulin omission behaviors and therefore require careful supervision. Conversely, people using CGM and hybrid closed-loop systems may be better positioned to adopt structured diet and exercise programs by reducing hypoglycemia burden and enabling safer insulin adjustments around activity. Clinically, the most pragmatic approach is individualized, preference-sensitive dietary counseling and gradual activity increases, coupled with structured insulin adjustment and hypoglycemia/DKA risk mitigation, while emphasizing the need for longer-term, T1DM-specific RCTs that stratify by age, adiposity phenotype, and technology use to define both efficacy and safety.

### Pharmacological Management of Obesity

Currently, several pharmacological agents are approved for the management of obesity, and additional therapies are anticipated in the coming years [73]. Although T1DM is not a formal contraindication to the use of approved anti-obesity medications, individuals with T1DM have largely been excluded from key obesity trials, resulting in limited T1DM-specific efficacy and safety data. Population-level registry data have shown that up to 79% of adults with T1DM do not achieve recommended glycemic targets [74, 75], and the prevalence of overweight and obesity is high, as mentioned above [20, 21, 23, 24]. Adjunct therapies may provide a useful strategy for weight management in people with T1DM. By targeting complementary pathways, improving insulin sensitivity (e.g., metformin), delaying gastric emptying and suppressing glucagon and appetite (e.g., pramlintide, GLP-1 medicines), enhancing incretin signaling (GLP-1 medicines) these agents can reduce insulin requirements while supporting tighter glycemic control [2, 40, 76]. In addition, GLP-1 medicines exert pleiotropic effects, including weight reduction and favorable cardiometabolic actions [77–79]. Although most were developed primarily to improve glycemic outcomes, several have also demonstrated clinically relevant benefits for weight management. RCTs of GLP-1 medicines as adjuncts to insulin in adults with T1DM are summarized in Table 2.

**Table 2:** Randomized controlled trials of GLP1-medicines in adults with T1DM

Trial	Study Design & Population	Intervention	Key Efficacy Outcomes	Safety Signals
Liraglutide				
Mathieu et al. 2016 [80] *ADJUNCT ONE*	RCT, 52 weeks Adults with T1DM +BMI ≥ 20 kg/m^2^ (*n* = 1,398)	Liraglutide 0.6/1.2/1.8 mg once daily vs. placebo	HbA1c reduction (1.8 mg −0.54% vs. placebo − 0.34% *p* < 0.001) Weight loss with 1.8 mg −4.9 kg TDD − 8% with 1.8 mg	Higher rates of symptomatic hypoglycemia across liraglutide dose groups [1.8 mg: 1.31 (1.07–1.59)] Higher rates of hyperglycemia with ketosis with liraglutide 1.8 mg: [2.22 (1.13–4.34)]
Ahren et al. 2016 [81] *ADJUNCT TWO*	RCT, 26 weeks Adults with T1DM +BMI ≥ 20 kg/m^2^ (*n* = 835)	Liraglutide 0.6/1.2/1.8 mg once daily vs. placebo	HbA1c reduction (1.8 mg −0.33% vs. placebo 0.01%) Weight loss with 1.8 −5.1 kg TDD − 10% Improved quality of life	Higher symptomatic hypoglycemia (1.2 mg: 21.3 vs. 16.6 events/patient/year; *p* = 0.03) Higher rates of hyperglycemia with ketosis with 1.8 mg (0.5 vs. 0.1 events/patient/year; *p* = 0.01)
Dejgaard et al. 2016 [82] *Lira-1*	RCT, 24 weeks Adults with T1DM + BMI > 25 kg/m² (*n* = 100)	Liraglutide 1.8 mg once daily vs. placebo	No significant between-group difference in HbA1c Weight loss − 6.8 kg vs. placebo Bolus insulin − 5.8 IU/day	Lower hypoglycemia event rates with liraglutide [0.82 (0.74–0.90)]
Kuhadiya et al. 2016 [83]	RCT, 12 weeks Adults with T1DM (*n* = 72)	Liraglutide 0.6/1.2/1.8 mg once daily vs. placebo	Mean weekly average glucose decreased with 1.2 and 1.8 mg (both − 10 mg/dL; *p* < 0.0001) HbA1c decreased significantly only with 1.2 mg (− 0.78%, *p* < 0.01) TDD decreased with higher doses Dose-dependent weight loss (5 kg with 1.2/1.8 mg)	Hypoglycemia incidence did not differ between groups
Dejgaard et al. 2020 [84] *Lira Pump trial*	RCT, 26 weeks Adults with T1DM +BMI [111] > 25 kg/m^2^ on CSII (*n* = 44)	Liraglutide 1.8 mg once daily vs. placebo	HbA1c −0.5% vs. placebo + 0.2% (between-group 0.7%; *p* < 0.001) Weight − 6.8 kg (between-group − 6.3 kg, *p* < 0.001) TDD − 8 units/day (*p* = 0.008; mainly bolus; weight-adjusted bolus/basal NS) TIR increased with liraglutide: 57% versus 45% (*p* = 0.044)	Hypoglycemia risk did not differ between groups
Ghanim et al. 2020 [85]	RCT, 26 weeks Adults with T1DM +BMI ≥ 25 kg/m^2^ (*n* = 84)	Liraglutide 1.8 mg once daily vs. placebo	HbA1c −0.41%, no significant placebo-adjusted difference (−0.25%; *p* = 0.14) TIR increased (*p* = 0.015) Weight − 4.2 kg driven mainly by fat mass loss (− 3.4 kg; *p* = 0.01 vs. placebo) No change in lean mass	Hypoglycemia risk did not differ between groups
Semaglutide				
Shah et al. 2025 [86] *ADJUST-T1D*	RCT, 26 weeks Adults with T1DM +BMI ≥ 30 kg/m^2^ (*n* = 72)	Semaglutide 1 mg once weekly vs. placebo	Primary composite glycemic-and-weight endpoint* achieved in 36% with semaglutide vs. 0% with placebo [between-group difference 36% points (95% CI 20.6–52.2); *p* < 0.001] HbA1c −0.3% TIR + 8.8% points vs. placebo Weight − 8.8 kg vs. placebo	Severe hypoglycemia similar (2 events/group) No DKA
Pasqua et al. 2025 [87]	RCT with crossover design, 4 weeks Adults with T1DM +BMI > 21 kg/m^2^ (*n* = 28)	Semaglutide 1 mg once weekly vs. placebo	Semaglutide increased TIR vs. placebo (+ 4.8% points; *p* = 0.006) Weight − 5.3 kg vs. placebo (*p* < 0.001, −5.1% from baseline)	No increase in time < 70 mg/dL (*p* = 0.19) or < 54 mg/dL (*p* = 0.65) No DKA or severe hypoglycemia 2 episodes of recurrent euglycemic ketosis without acidosis with semaglutide
Tirzepatide				
Snaith et al. 2025 [94]	RCT, 12 weeks Adults with T1DM +BMI > 30 kg/m^2^ (*n* = 24)	Tirzepatide once weekly vs. placebo	Weight change − 10.3 kg vs. −0.7 kg (ETD − 8.7 kg, *p* < 0.0001) All achieved ≥ 5% weight loss with tirzepatide ≥ 10% weight loss achieved in 45% with tirzepatide vs. 0% with placebo HbA1c −0.4% (*p* = 0.05) TDD − 24.2 vs. −0.3 units/day (−35.1%; *p* = 0.0002)	No significant adverse effects

*Achievement of CGM time in range (70–180 mg/dL) > 70%, time below range (< 70 mg/dL) < 4%, and ≥ 5% weight loss

*T1DM* type 1 diabetes, *RCT* randomized controlled trial, *BMI* body mass index, *TDD* total daily insulin dose, *CI* confidence interval, *CSII* continuous subcutaneous insulin infusion, *DKA* diabetic ketoacidosis, *TIR* time in range, *ETD* estimated treatment difference, *NS* not significant, *TIR* time in range

#### Liraglutide

Liraglutide is the most extensively studied GLP-1 medicine in adults with T1DM. The ADJUNCT ONE trial is the largest and longer RCT to date evaluating a GLP-1 medicine as an adjunct to insulin therapy in adults with T1DM [80]. In this 52-week RCT, 1,398 adults with T1DM and BMI ≥ 20 kg/m^2^ were randomized to once-daily liraglutide (1.8, 1.2, or 0.6 mg) or placebo. The primary endpoint of this study was to examine the efficacy of liraglutide on glycemic control, the reduction in TDD and body weight loss compared with placebo. HbA1c was reduced in a modest, dose-dependent manner; mean change was − 0.34% with placebo versus − 0.54% with liraglutide 1.8 mg (*p* < 0.001). In the 1.8 mg group, participants achieved a mean weight loss of 4.9 kg (95% CI: −5.7 to −4.2) and an 8% (95% CI: 0.88–0.96) reduction in TDD (primarily bolus insulin). Regarding safety, symptomatic hypoglycemia occurred more frequently across all liraglutide dose groups, including the 1.8 mg arm [estimated rate ratio (95% CI): 1.31 (1.07–1.59)]. In addition, hyperglycemia with ketosis was significantly increased with liraglutide 1.8 mg only [event rate ratio (95% CI): 2.22 (1.13–4.34)].

The ADJUNCT TWO trial was a 26-week, RCT evaluating once-daily liraglutide (1.8, 1.2, or 0.6 mg) as adjunct therapy to insulin in adults with T1DM and BMI ≥ 20 kg/m^2^, using an individually capped insulin regimen [81]. Overall, 835 participants were randomized. The primary endpoint was the change in HbA1c. At week 26, liraglutide significantly reduced HbA1c versus placebo (mean change: −0.33% with 1.8 mg compared with 0.01% with placebo) and produced clinically meaningful weight loss (−5.1 kg with 1.8 mg versus − 0.2 kg with placebo) [81]. These effects were accompanied by reductions in TDD [1.8 mg: 0.90 (95% CI): 0.86–0.93], and improvements in quality of life. However, safety signals were observed, including higher rates of symptomatic hypoglycemia, particularly with the 1.2 mg dose (21.3 vs. 16.6 events/patient/year; *p* = 0.03) and an increased incidence of hyperglycemia with ketosis with liraglutide 1.8 mg compared with placebo (0.5 vs. 0.1 events/patient/year; *p* = 0.01). Hypoglycemia rates may have been influenced by trial design, with the capped-insulin regimen in ADJUNCT TWO potentially contributing to a more pronounced hypoglycemia signal compared with the treat-to-target approach in ADJUNCT ONE [80, 81].

In the Lira-1 trial, adults with T1DM and BMI > 25 kg/m² were randomized to adjunctive liraglutide 1.8 mg or placebo for 24 weeks [82]. Change in HbA1c was the primary endpoint. Α total of 100 participants were enrolled (50 per group). HbA1c decreased in both groups, with no significant between-group difference. Liraglutide achieved clinically meaningful weight loss (−6.8 kg vs. placebo) and reduced bolus insulin requirements (−5.8 IU/day). Notably, liraglutide was also associated with fewer hypoglycemic events (incident rate ratio 0.82, 95% CI 0.74–0.90). Liraglutide produced a clinically meaningful reduction in body weight (−6.8 kg vs. placebo) and was accompanied by lower bolus insulin needs (−5.8 IU/day) [82]. Notably, liraglutide was also associated with fewer hypoglycemic events with an incident rate ratio of 0.82 (95% CI: 0.74–0.90).

In a 12-week RCT, 72 adults with T1DM were allocated to once-daily liraglutide (0.6, 1.2, or 1.8 mg; *n* = 54 overall) or placebo (*n* = 18) [83]. The primary endpoint was the change in mean weekly blood glucose. Mean weekly average glucose decreased significantly with liraglutide 1.2 mg and 1.8 mg (both − 10 mg/dL, *p* < 0.0001), with nonsignificant change in the 0.6 mg or placebo groups. HbA1c decreased significantly only with liraglutide 1.2 mg (−0.78%, *p* < 0.01), whereas changes were not significant with 1.8, 0.6 mg or placebo. TDD declined significantly in the 1.2 mg and 1.8 mg groups (*p* < 0.05). Weight loss was dose-dependent, reaching 5 kg with 1.2 mg and 1.8 mg (*p* < 0.05) versus no change with placebo. Hypoglycemia incidence was not different between the groups.

In a 26-week RCT (Lira Pump trial), 44 adults with overweight or obesity and T1DM on suboptimal glycemic control using continuous subcutaneous insulin infusion were assigned to liraglutide 1.8 mg or placebo [84]. The primary endpoint was change in HbA1c. Liraglutide reduced HbA1c by 0.5% from a baseline of 8.2%, whereas placebo produced a non-significant increase of 0.2% from a baseline of 8.1%, yielding a between-group difference of 0.7% (*p* < 0.001). Liraglutide reduced body weight by 6.8 kg at 26 weeks (*p* < 0.001), yielding a between-group difference of −6.3 kg (*p* < 0.001). TDD decreased by 8 units/day (*p* = 0.008), driven mainly by lower bolus insulin. However, after adjustment for body weight, no significant changes were observed in total bolus or basal insulin doses in either group over the study period. Participants treated with liraglutide had more time in range (TIR) compared with placebo, 57% versus 45% (*p* = 0.044). Hypoglycemia risk did not differ between groups.

In another 26-week RCT, 84 adults with T1DM and BMI ≥ 25 kg/m² were randomized to placebo or liraglutide 1.8 mg/day [85]. The primary endpoint was the effect of liraglutide on mean HbA1c. Secondary endpoints included body composition outcomes. Subcutaneous adipose tissue biopsies, DXA and MRI were performed before and at the end of treatment. Liraglutide reduced HbA1c from 7.96% to 7.55% (−0.41%; *p* = 0.001 vs. baseline); however, the placebo-adjusted difference at 26 weeks was not significant (−0.25%, *p* = 0.14). There was no increase in hypoglycemia, while the time spent in normal glycaemia increased (*p* = 0.015) and time spent in hyperglycemia decreased (*p* = 0.019). Liraglutide was associated with significant weight loss (4.2 ± 0.6 kg), driven predominantly by a reduction in fat mass (−3.4 ± 0.6 kg; *p* = 0.01 vs. placebo), accounting for 82% of total weight loss, with no significant change in lean mass as measured by DEXA. Notably, baseline body weight was not associated with the magnitude of weight or fat mass loss. Fat mass loss was partly driven by a reduction in visceral adipose tissue on MRI (−0.52 ± 0.14 L, *p* = 0.005 vs. placebo).

Overall, across RCTs in adults with T1DM, liraglutide consistently produces clinically meaningful weight loss and modest reductions in insulin requirements, with small improvements in glycemic indices in selected settings. Notably, these trials evaluated liraglutide up to 1.8 mg/day, whereas higher doses are used for obesity management (up to 3.0 mg). Therefore, the magnitude of weight-loss efficacy and the safety profile, particularly regarding hypoglycemia and hyperglycemia with ketosis, at obesity-dose liraglutide in T1DM remain uncertain. In all cases, potential benefits should be weighed against dose-dependent safety signals, highlighting the need for careful patient selection, structured insulin adjustment, and close monitoring.

#### Semaglutide

In the ADJUST-T1D RCT, 72 adults with T1DM and BMI ≥ 30 kg/m² were randomized to semaglutide 1 mg or placebo for 26 weeks [86]. The primary composite endpoint was achievement of TIR (70–180 mg/dL) > 70%, time-below-range (< 70 mg/dL) < 4%, and ≥ 5% weight loss. A significantly greater proportion of participants receiving semaglutide met the composite endpoint compared with placebo [36% vs. 0%; between-group difference (95% CI): 36% points (20.6–52.2), *p* < 0.001]. Semaglutide also improved key secondary outcomes versus placebo, including HbA1c (−0.3%), time-in-range (+ 8.8% points), and body weight (−8.8 kg). Severe hypoglycemia occurred at similar frequency (two events in each group), and no DKA was reported [86].

In a RCT with a crossover design, semaglutide was evaluated as an adjunct to automated insulin delivery (AID) therapy in adults with T1DM. During each intervention period, participants were titrated over 11 weeks to semaglutide up to 1 mg (or the maximum tolerated dose) or placebo, followed by 4 weeks of AID use [87]. The primary outcome was the percentage of TIR during the final 4 weeks of each intervention. Overall, 28 participants were randomized and 24 completed the study. Semaglutide significantly increased TIR compared with placebo by a mean 4.8% points (*p* = 0.006), without increasing time below 70 mg/dL (*p* = 0.19) or below 54 mg/dL (*p* = 0.65). Body weight decreased by 5.3 kg with semaglutide versus placebo (*p* < 0.001), corresponding to a −5.1% relative change from baseline. No DKA or severe hypoglycemia occurred during either intervention; however, two episodes of recurrent euglycemic ketosis without acidosis were reported during semaglutide treatment [87].

Real-world evidence has also been reported in adults with T1DM living with obesity receiving adjunct semaglutide [88]. In a 12-month observational study of 42 adults with overweight/obesity and stable baseline control, once-weekly semaglutide was associated with substantial weight loss (−13.3%, *p* < 0.001), a modest HbA1c reduction (−0.4%, *p* < 0.001), and lower TDD requirements (−13.6%, *p* < 0.001), although CGM metrics did not change in those with available data. In a subgroup with serial liver assessment (*n* = 23), MASLD prevalence fell from 82.6% to 30.4% (*p* < 0.001), controlled attenuation parameter decreased (− 45 dB/m), and significant fibrosis (LSM > 8 kPa) declined (20.6% to 4.5%, *p* < 0.001) [88].

In another small study of 11 individuals with T1DM receiving a lower dose of semaglutide (0.5 mg once weekly), promising outcomes were reported, including an 10.6% reduction in body weight (mean loss, 8.8 kg) [89]. TDD decreased from a mean of 45.6 to 38.5 U/day, driven by proportional reductions in both basal and bolus insulin [89].

Ongoing trials are evaluating whether semaglutide can improve cardiometabolic risk markers in people with T1DM. The phase 2 RESET1 RCT will enroll 60 adults with T1DM (age 25–70 years; duration ≥ 2 years) living with overweight (BMI ≥ 25 kg/m²), HbA1c ≥ 7%, and at least one CV risk factor, and will compare semaglutide up to 1.0 mg weekly versus placebo over 26 weeks [90]. The primary endpoint is carotid-femoral pulse wave velocity (arterial stiffness), with mechanistic assessments including insulin sensitivity by hyperinsulinemic-euglycemic clamp and incretin/pancreatic hormone responses during a mixed-meal tolerance test. Another ongoing study is T1-DISCO (Type 1 Diabetes Impacts of Semaglutide on Cardiovascular Outcomes), which is evaluating semaglutide’s effects on arterial stiffness, kidney function, and insulin sensitivity in adults with T1DM [91]. Key vascular outcomes include changes in ascending aortic pulse wave velocity as a measure of central arterial stiffness and carotid-femoral and carotid-radial pulse wave velocity as measures of peripheral arterial stiffness (all assessed from baseline to month 8). Secondary outcomes include change in insulin and change in renal vascular resistance. In addition, SEMA-AP trial (Weekly Subcutaneous Semaglutide as an Adjunct to Closed-loop Therapy in T1DM Care), is evaluating whether adding semaglutide to an automated insulin delivery system can further improve glycemic control in T1DM [92]. Participants use a closed-loop insulin system and are randomized to weekly semaglutide (titrated to the maximum tolerated dose) versus placebo. The primary outcome is CGM time in range over 4 weeks, comparing semaglutide versus placebo while on closed-loop therapy. Furthermore, RT1D (Trial of Semaglutide for Diabetic Kidney Disease in T1DM) is a 26-week, RCT evaluating semaglutide’s renal effects in adults with T1DM [93]. The primary endpoint is kidney oxygenation assessed by MRI (cortical R2*), with key secondary outcomes including urine albumin-to-creatinine ratio and eGFR. Real-time CGM is used to support comparable glycemic control between groups while also assessing glycemic effects and safety (including insulin dose, severe hypoglycemia, and DKA).

Overall, these early observational data suggest that adjunctive semaglutide may facilitate meaningful weight loss in people with T1DM living with overweight or obesity. Importantly, several ongoing RCTs are now evaluating semaglutide across cardiometabolic, vascular, glycemic, and renal endpoints, which should better define both efficacy and safety. Until these data mature, unresolved safety considerations, including hypoglycemia and ketosis/DKA, support cautious use and reinforce the need for adequately powered, longer-term trials in T1DM.

#### Tirzepatide

In a 12-week, phase 2 RCT, tirzepatide was evaluated in adults with T1DM and BMI > 30 kg/m². Participants were randomized to once-weekly tirzepatide (2.5 mg for 4 weeks, then 5.0 mg for 8 weeks) or placebo [94]. The primary endpoint was change in body weight at week 12. Of 24 randomized participants, 22 completed the study. At 12 weeks, mean weight change was − 10.3 kg with tirzepatide versus − 0.7 kg with placebo, yielding an estimated treatment difference of −8.7 kg (*p* < 0.0001), corresponding to 8.8% weight loss. All participants receiving tirzepatide achieved ≥ 5% weight loss and 45% achieved ≥ 10%, compared with 9% and 0% with placebo. Tirzepatide also improved HbA1c (mean difference − 0.4% vs. placebo; *p* = 0.05) and reduced TDD requirements (−24.2 vs. −0.3 units/day; −35.1% vs. placebo; *p* = 0.0002), with no significant adverse events reported [94].

Observational evidence on tirzepatide in T1DM has also been presented in a meta-analysis of six studies [95]. Across pooled analyses, tirzepatide was associated with a mean HbA1c reduction of 0.68% (95% CI −0.72 to −0.64, I²=20.3%, *p* < 0.0001) and a mean weight reduction of 12.36 kg (95% CI −17.87 to −6.85, I²=96.8%, *p* = 0.0015) when added to insulin therapy. Additional efficacy signals included reductions in BMI of 6.74 kg/m² (*p* < 0.0001) and TDD of 25.9 units (*p* < 0.0001). With respect to CGM metrics, tirzepatide was associated with an increase in TIR of 14.8% (*p* = 0.0037), a mild but significant increase in time-below-range of 0.54% (*p* < 0.0001), and a reduction in time-above-range of 15.39% (*p* = 0.016) [95].

An additional ongoing trial, SURPASS-T1D-1, is a phase 3, RCT evaluating tirzepatide once weekly versus placebo in adults with T1DM living with overweight or obesity [96]. Total participation is approximately 49 weeks. Key outcomes include change in HbA1c and change in body weight, with CGM-based efficacy also assessed, including TIR (assessed around week 40).

Overall, most GLP-1 RA studies in T1DM have prioritized glycemic endpoints (e.g., HbA1c and CGM metrics) as the primary outcome, with weight loss typically assessed as a secondary endpoint. Moreover, several trials enrolled broadly defined populations, often including people with normal BMI, which limits the ability to quantify the additional clinical impact of weight loss in those living with overweight or obesity. Early data with tirzepatide are encouraging, but the phase 2 RCT was short in duration and included a small sample. Larger, longer-term RCTs are needed to define efficacy, safety, and the populations most likely to benefit. Importantly, the current evidence for both semaglutide and tirzepatide in T1DM remains emerging and incomplete, with key unknowns including durability of benefit, long-term ketosis/DKA risk in the setting of insulin dose reduction, severe hypoglycemia risk in broader practice, effects on microvascular and CV endpoints, and safety and efficacy in youth.

When considering adjunct GLP-1-based therapy in people with T1DM living with overweight or obesity, practical safety measures are essential. Insulin dose reductions should be individualized and conservative, typically beginning with modest reductions in both prandial and basal insulin, followed by stepwise titration guided by close CGM review, with the dual aim of minimizing hypoglycemia while avoiding excessive insulin withdrawal. Patients should also receive clear instructions on ketone monitoring, particularly during intercurrent illness, persistent hyperglycemia, vomiting or reduced carbohydrate intake, unexplained malaise, or after insulin dose reductions. Rising ketones should prompt corrective insulin, hydration, carbohydrate intake as appropriate, and urgent medical assessment when ketones are moderate-high or symptoms suggest evolving DKA. In general, these agents should be avoided in individuals at high risk for ketosis/DKA or unsafe insulin use, including those with prior DKA or recurrent ketosis, very low-carbohydrate/ketogenic diets, inconsistent insulin administration or high pump-failure risk, eating disorder behaviors/intentional insulin omission, low BMI or frailty.

#### Metformin

A meta-analysis of 19 RCTs (*n* = 1,540, mean BMI 27.5 kg/m²) found that adjunctive metformin in T1DM was associated with a modest reduction in HbA1c of 0.26% [97]. In the same analysis, metformin was also linked to lower TDD requirements (SMD − 0.81, I^2^: 91.6, *p* < 0.001) alongside decreases in body weight (−2.24 kg). Gastrointestinal adverse effects were more common with metformin than placebo [RR (95% CI): 2.01 (1.35–3.00.35.00)], while no differences were observed in severe hypoglycemia, lactic acidosis, or DKA [97].

A recent 26-week RCT evaluated metformin (1500 mg/day) as an adjunct to target insulin resistance in adults with T1DM (*n* = 20 per arm) using a two-step hyperinsulinemic-euglycemic clamp [98]. The primary outcome was change in endogenous glucose production during the low-dose phase of the clamp. Although participants with T1DM exhibited tissue-specific insulin resistance at baseline versus non-diabetic controls, metformin did not improve hepatic insulin resistance, with no between-group difference in change in endogenous glucose production during the low-dose clamp phase (mean difference 0.2 µmol/kg fat-free mass/min; 95% CI −0.4 to 0.8; *p* = 0.53) [98]. These findings suggest that, at least over 26 weeks, metformin’s potential clinical benefits in T1DM may not be mediated by improvements in hepatic insulin sensitivity.

#### Pramlintide

Pramlintide, a synthetic analogue of human amylin, is the only adjunctive glucose-lowering therapy approved for use in people with T1DM in the U.S [2, 99]. Amylin is a naturally occurring hormone that is co-secreted with insulin by pancreatic β-cells.

In a 52-week RCT, 651 adults with T1DM were randomized to mealtime placebo or pramlintide at varying doses, added to background insulin therapy [100]. Compared with placebo (HbA1c −0.04%), pramlintide at 60 µg three times daily or four times daily produced significant HbA1c reductions [−0.29% (*p* < 0.011) and − 0.34% (*p* < 0.001), respectively], with approximately threefold more pramlintide-treated participants achieving HbA1c < 7%. These glycemic improvements occurred without an increase in concomitant insulin use and were accompanied by modest weight benefits: body weight decreased by 0.4 kg with pramlintide three times a day (*p* < 0.027) versus a 0.8 kg gain with placebo. Transient mild-to-moderate nausea was the most frequently reported adverse event. However, pramlintide use was also associated with an approximately fourfold higher risk of severe hypoglycemia [100]. The required injection frequency and higher cost remain key barriers to the widespread uptake of pramlintide in people living with T1DM [2, 99].

#### SGLT Inhibitors (SGLT2 and Dual SGLT1/2 Inhibitors)

Sodium-glucose cotransporter-2 (SGLT2) inhibitors they are approved for the treatment of T2DM, heart failure and chronic kidney disease [101]. In DEPICT-1, a phase 3, RCT (*n* = 833), dapagliflozin (5 mg or 10 mg) was evaluated as an adjunct to adjustable insulin in people with T1DM and inadequate glycemic control (HbA1c 7.5–10.5%) [102]. Over 52 weeks, both doses achieved modest but clinically meaningful HbA1c reductions versus placebo (−0.33 to −0.36%) and significant weight loss (−3.0% to −4.5%), with similar hypoglycemia rates across groups. However, adjudicated DKA occurred more frequently with dapagliflozin (3.4–4.0.4.0%) than placebo (1.9%), indicating improved glycemic and weight outcomes at the cost of increased DKA risk [102].

In the DEPICT-2 trial, results were reported from a similar design in patients from Asia, predominantly Japan [103]. In this phase 3 RCT, 813 people with T1DM were randomized to dapagliflozin 5 mg, 10 mg, or placebo. Compared with placebo, dapagliflozin led to mean percentage body weight reductions of 4.4% (5 mg) and 4.9% (10 mg), alongside modest HbA1c reductions of 0.20% and 0.25%, respectively. As in DEPICT-1, DKA was more frequent with dapagliflozin than placebo (4.1% with 5 mg, 3.7% with 10 mg, and 0.4% with placebo). Compared with placebo, dapagliflozin led to mean percentage body weight reductions of 4.42% (5 mg) and 4.86% (10 mg), alongside modest HbA1c reductions of 0.20% and 0.25%, respectively. As in DEPICT-1, DKA was more frequent with dapagliflozin than placebo (4.1% with 5 mg, 3.7% with 10 mg, and 0.4% with placebo) [103].

In a post-hoc analysis of the DEPICT-1 and DEPICT-2 trials in adults with T1DM and BMI ≥ 27 kg/m², adjudicated DKA occurred in 5 (1.7%) participants receiving dapagliflozin versus 3 (1.0%) receiving placebo [104]. Notably, fewer definite DKA events were observed in this BMI ≥ 27 kg/m² subgroup than in the overall pooled DEPICT population, suggesting a lower DKA risk compared with participants with BMI < 27 kg/m².

The Empagliflozin as Adjunctive to inSulin thErapy (EASE) program comprised two phase 3 RCTs: EASE-2 (52 weeks), which randomized participants to empagliflozin 10 mg (*n* = 243), 25 mg (*n* = 244), or placebo (*n* = 243), and EASE-3 (26 weeks), which evaluated empagliflozin 2.5 mg (*n* = 241), 10 mg (*n* = 248), 25 mg (*n* = 245), or placebo (*n* = 241). Overall, 1,707 people with T1DM received empagliflozin as an adjunct to insulin [105]. Across doses (2.5–25 mg), empagliflozin reduced body weight (−1.8 to −3.4 kg) and improved HbA1c and insulin dose requirements, but DKA occurred more frequently with 10 mg (4.3%) and 25 mg (3.3%) compared with 2.5 mg (0.8%) and placebo (1.2%) [105].

In the phase 3, inTandem3 RCT, 1,402 adults with T1DM receiving insulin therapy were randomized to sotagliflozin (dual SGLT1/2 inhibitor) 400 mg/day or placebo for 24 weeks [106]. A greater proportion of participants receiving sotagliflozin achieved the composite primary endpoint of HbA1c < 7.0% without severe hypoglycemia or DKA (28.6% vs. 15.2%; *p* < 0.001). Sotagliflozin also improved secondary outcomes, including greater reductions in HbA1c (between-group difference − 0.46% points), body weight (−2.98 kg), systolic blood pressure (−3.5 mmHg), and mean daily bolus insulin dose (−2.8 units/day) (all *p* ≤ 0.002). Severe hypoglycemia rates were similar between groups (3.0% vs. 2.4%), and documented hypoglycemia ≤ 55 mg/dL was lower with sotagliflozin. However, DKA occurred more frequently with sotagliflozin than placebo (3.0% vs. 0.6%) [106].

In the 52-week phase 3 inTandem1 RCT in North American adults with T1DM (*n* = 793), sotagliflozin 200 mg or 400 mg added to optimized insulin reduced HbA1c versus placebo at 24 weeks (placebo-adjusted − 0.36% and − 0.41%) and 52 weeks (−0.25% and − 0.31%; all *p* < 0.001) and promoted weight loss (−4.32 kg with 400 mg at 52 weeks) alongside lower insulin requirements [107]. However, genital mycotic infections and diarrhea were more common; adjudicated DKA was increased (3.4–4.2% vs. 0.4%), underscoring the need for careful selection and DKA risk mitigation when considering adjunct SGLT inhibition in T1DM [107].

In the 52-week, phase 3 inTandem2 RCT in adults with T1DM (after a 6-week insulin optimization run-in), sotagliflozin 200 mg or 400 mg added to insulin improved glycemic outcomes versus placebo [108]. Placebo-adjusted HbA1c reductions at 24 weeks were − 0.37% and − 0.35% (*p* < 0.001) and were maintained through 52 weeks. By week 52, a greater proportion of sotagliflozin-treated participants met a composite endpoint (HbA1c < 7.0% with no severe hypoglycemia and no DKA) compared with placebo (26% vs. 14%; *p* ≤ 0.001), alongside reductions in weight (−2.92 kg), and TDD (−8.2%). CGM data also suggested improved postprandial glucose and up to 3 additional hours/day in range. However, DKA occurred more frequently with sotagliflozin (2.3–3.4% vs. 0% with placebo), despite fewer documented and severe hypoglycemia events [108].

In pooled data from the inTandem1 and inTandem2 sotagliflozin RCTs in T1DM, adjudicated DKA event rates (exposure-adjusted incidence rates per 100 patient-years) were numerically higher in those with BMI < 27 kg/m² versus BMI ≥ 27 kg/m² [109]. BMI < 27 kg/m²: placebo 0, sotagliflozin 200 mg 3.5 (95% CI 0.9–6.1) and 400 mg 4.9 (1.7–8.0.7.0); BMI ≥ 27 kg/m²: placebo 0.4 (0.0–1.9.0.9), sotagliflozin 200 mg 2.9 (0.8–4.8) and 400 mg 3.8 (1.5–6.0.5.0) [109].

Across trials, SGLT inhibitors (SGLT2 and dual SGLT1/2 inhibitors) used as adjuncts to insulin in T1DM have consistently been associated with an increased risk of DKA versus placebo, despite modest improvements in HbA1c. Importantly, the accompanying reductions in body weight are clinically meaningful, supporting a potential role in carefully selected individuals living with overweight or obesity. Notably, post-hoc and pooled analyses indicate that DKA risk varies by BMI, with fewer DKA events reported in participants with BMI ≥ 27 kg/m², underscoring the need for careful patient selection and structured DKA risk-mitigation strategies when considering adjunct SGLT inhibition in T1DM.

#### Other Drugs

Orlistat is an approved anti-obesity medication that acts as a reversible gastrointestinal lipase inhibitor, reducing dietary fat absorption [99]. Naltrexone-bupropion is also approved for the management of obesity [99]. However, for both agents, clinical trial evidence evaluating safety and efficacy in people living with T1DM remains lacking [99].

### Bariatric Surgery

Bariatric procedures were historically categorized as “restrictive” or “malabsorptive” based on their presumed primary mechanism [110]. However, this classification is now largely outdated because each operation exerts weight-loss and metabolic effects through multiple, overlapping pathways. Roux-en-Y gastric bypass and vertical sleeve gastrectomy are the most commonly performed operations. Notably, most evidence for these techniques comes from studies in T2DM, whereas data in T1DM are largely retrospective, involve small cohorts, and include heterogeneous patient populations [111]. As bariatric procedures and their mechanisms have been described in detail previously [111], we focus here on the most recent data and the outcomes and safety considerations most relevant to T1DM.

The majority of safety and efficacy outcomes of bariatric surgery in T1DM are published with small sample sizes [111]. Overall, bariatric procedures have been associated with substantial and sustained reductions in BMI, TDD, and improvements have been reported in obesity-related comorbidities, including hypertension and dyslipidaemia [111]. In contrast, effects on glycemic control appear modest and are frequently not statistically significant [111]. Importantly, safety concerns have been raised, as peri-operative and post-operative adverse events often relate to metabolic instability, including DKA, severe hypoglycemia, and increased glucose variability. However, the conduct of large-scale prospective trials in this population is challenging, given that bariatric surgery is performed relatively infrequently in people with T1DM [111]. Accordingly, prospective studies are warranted to better delineate the long-term efficacy and safety of bariatric surgery in T1DM, but achieving adequate sample sizes will likely require multicentre, international collaboration [2].

## Conclusion

Overweight and obesity are now common in people with T1DM. Available epidemiological and genetic data suggest a bidirectional relationship. Greater adiposity may increase the risk of T1DM and/or accelerate disease progression in susceptible individuals. Conversely, weight gain in established T1DM is multifactorial. It reflects the obesogenic environment, insulin intensification and hypoglycemia-related behaviors, insulin resistance, and overlapping familial/genetic and hormonal determinants.

T1DM-specific evidence for obesity treatments remains comparatively limited and heterogeneous. Lifestyle interventions can support weight loss but require careful individualization in T1DM to minimize hypoglycemia and ketosis risk. GLP-1 therapies show early signals of weight loss and insulin-sparing effects in selected populations, but uncertainty remains regarding long-term safety (including hypoglycemia and ketosis/DKA risk), durability, and generalizability. Bariatric surgery can produce substantial weight loss and reduce insulin requirements, yet glycemic benefits are often modest and metabolic complications (including hypoglycemia and DKA) remain important considerations.

Future work should prioritize adequately powered, longer-duration RCTs specifically enrolling people with T1DM to evaluate efficacy, safety, and durability of obesity interventions, particularly incretin-based and combination pharmacotherapies. Trials should assess clinically meaningful outcomes beyond weight loss, including CGM-derived metrics, insulin requirements, hypoglycemia and DKA, CV and renal endpoints, and quality of life. Mechanistic studies are also needed to better define obesity phenotypes in T1DM, incorporating body composition and fat distribution, insulin resistance, genetics, and hormonal regulation, to support more personalized approaches. Finally, implementation research is required to determine how emerging therapies can be safely and equitably integrated into routine T1DM care across diverse populations and life stages. Until such data are available, obesity management in T1DM should be individualized, with careful balancing of potential benefits and risks and close monitoring.

## Key References

- Wei Y, Andersson T, Tuomi T, Nystrom T, Carlsson S. Adult-onset type 1 diabetes: predictors of major cardiovascular events and mortality. Eur Heart J. 2025;46(38):3776–3786. 10.1093/eurheartj/ehaf304.
  - This nationwide Swedish registry study shows that BMI, smoking, and HbA1c are key contributors to cardiovascular events in adult-onset T1DM.
- Nystrom T, Andersson Franko M, Ludvigsson J, Lind M, Persson M. Overweight or obesity, weight variability and the risk of retinopathy in type 1 diabetes. Diabetes Obes Metab. 2024;26(6):2509–2512. 10.1111/dom.15545.
  - This large Swedish registry cohort demonstrates that overweight/obesity in youth and young adults with T1DM is associated with an increased risk of retinopathy.
- Mathieu C, Zinman B, Hemmingsson JU, Woo V, Colman P, Christiansen E, et al. Efficacy and Safety of Liraglutide Added to Insulin Treatment in Type 1 Diabetes: The ADJUNCT ONE Treat-To-Target Randomized Trial. Diabetes Care. 2016;39(10):1702–1710. 10.2337/dc16-0691.
  - This is the largest RCT evaluating a GLP-1 receptor agonist (liraglutide) versus placebo in people with T1DM, providing key evidence on weight loss, insulin-sparing effects, and safety outcomes.
- Shah VN, Akturk HK, Kruger D, Ahmann A, Bhargava A, Bakoyannis G, et al. Semaglutide in Adults with Type 1 Diabetes and Obesity. NEJM Evid. 2025;4(8):EVIDoa2500173. 10.1056/EVIDoa2500173.
  - This RCT shows that adjunct semaglutide 1 mg improves CGM metrics and induces substantial weight loss in adults with T1DM and obesity, supporting a role for GLP-1 RA therapy beyond insulin in selected individuals.
- Snaith JR, Frampton R, Samocha-Bonet D, Greenfield JR. Tirzepatide in Adults With Type 1 Diabetes: A Phase 2 Randomized Placebo-Controlled Clinical Trial. Diabetes Care. 2025. 10.2337/dc25-2379.
  - This phase 2 RCT shows that tirzepatide induces substantial short-term weight loss with modest glycemic and insulin-sparing benefits in adults with T1DM and obesity.

## Data Availability

No datasets were generated or analysed during the current study.
